# Micrograin Superplasticity: Characteristics and Utilization

**DOI:** 10.3390/ma4071194

**Published:** 2011-07-01

**Authors:** Farghalli A. Mohamed

**Affiliations:** Department of Chemical Engineering and Materials Science, University of California, Irvine, CA 92697, USA; E-Mail: famohame@uci.edu; Tel.: +949-842-5807; Fax: +949-824-2451

**Keywords:** cavitation, cryomilling, dislocation activity, ductility, grain boundary sliding, high-strain rate superplasticity, impurity effect, micrograin superplasticity, nanocrystalline materials

## Abstract

Micrograin Superplasticity refers to the ability of fine-grained materials (1 µm < *d* < 10 μm, where *d* is the grain size) to exhibit extensive neck-free elongations during deformation at elevated temperatures. Over the past three decades, good progress has been made in rationalizing this phenomenon. The present paper provides a brief review on this progress in several areas that have been related to: (a) the mechanical characteristics of micrograin superplasticity and their origin; (b) the effect of impurity content and type on deformation behavior, boundary sliding, and cavitation during superplastic deformation; (c) the formation of cavity stringers; (d) dislocation activities and role during superplastic flow; and (e) the utilization of superplasticity.

## I. Introduction

**Figure 1:**
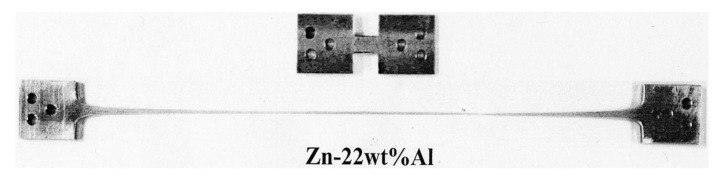
Ductility associated with micrograin superplasticity.

Superplasticity refers to the ability of fine-grained materials (1 μm < *d* < 10 μm, where d is the grain size) materials to exhibit extensive neck-free elongations during deformation at elevated temperatures (*T* > 0.5 *T_m_*, where *T_m_* is the melting point). An example that illustrates ductility associated with superplastic flow is given in Figure 1.

Considerable interest has developed in micrograin superplasticity [1,2,3]. This interest has arisen partly from a scientific viewpoint and partly from the increasing awareness that superplastic materials can be utilized in forming complex shapes in simple and inexpensive forming operations. There are two main advantages in utilizing superplastic materials for metal forming operations. First, large strains can be achieved without necking. Second, the stresses required for superplastic deformation are generally low.

The two basic requirements for the observation of micrograin superplasticity in materials are: (a) a temperature greater than about one-half of the melting points, *T_m_*, and (b) a fine and equiaxed grain size (<10 µm) that does not undergo significant growth during high temperature deformation. In addition to these two requirements, grain boundaries need to be mobile, high-angled and able to resist tensile separation. The requirement of a small grain size has resulted in the development of several superplastic alloys based on binary or ternary eutectic or eutectoid systems since grain growth is then inhibited by the presence of two or more phases.

Over the past four decades, significant progress has been made not only in understanding the origin of micrograin superplasticity but also in utilizing this phenomenon for structural applications. Accordingly, the purpose of this paper is to review some of the fundamental progress that has been made. In particular, the paper will focus on reviewing the following aspects of the fundamental progress: (a) the origin of the relation between stress and strain rate, (b) effect of impurity content and type on deformation and boundary sliding, (c) the mechanism by which cavity stringers are formed during superplastic deformation, (d) the role played by lattice dislocation during superplasticity. In addition, the paper will address some concepts that have introduced to exploit superplasticity for commercial forming of structural components.

## 2. Discussion

### 2.1. Mechanical Characteristics

**Figure 2:**
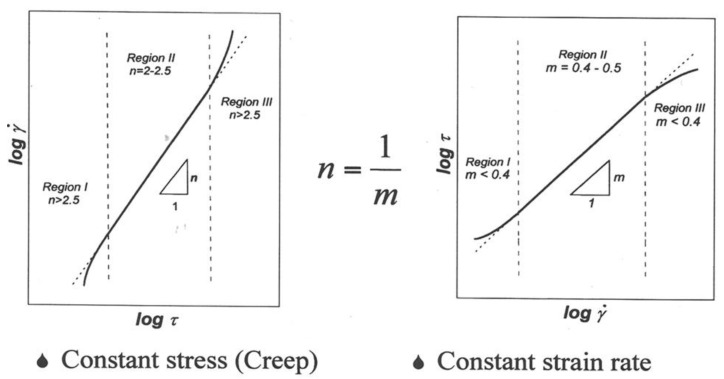

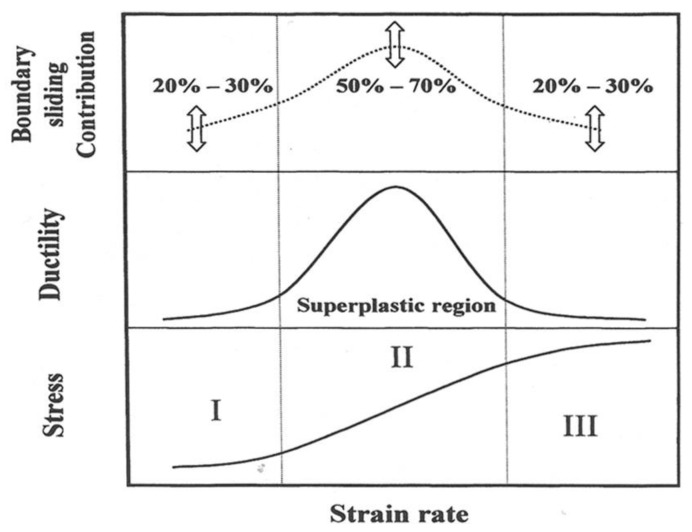
(**a**) Schematic representation regarding the relation between applied stress and strain rate for micrograin superplasticity; (**b**) A schematic representation for the characteristics of micrograin superplasticity.

Micrograin superplasticity is regarded as a creep phenomenon since it has been observed at temperatures at or above 0.5 of the melting point. Accordingly, in establishing the mechanical behavior of superplastic alloys, investigators extensively studied the following four relationships that define the basic deformation characteristics associated with a creep process: (a) the relationship between stress and strain rate, (b) the relationship between strain rate or stress and temperature, (c) the relationship between strain rate or stress and grain size, and (d) the relationship between strain contributed by boundary sliding and total strain. As a result of the studies on the aforementioned relationships, three findings are well documented. First, micrograin superplasticity is a diffusion-controlled process that can be represented by the following dimensionless equation [4,5]:

(1a) $$\frac{\dot{\gamma }kT}{D\;Gb}=A{\left(\frac{b}{d}\right)}^{s}{\left(\frac{\tau }{G}\right)}^{n}$$

with

(1b) $$D=D_{o}\;exp(-\frac{Q}{RT})$$

where $\dot{\gamma }$ is the shear creep rate, *k* is Boltzmann’s constant, *T* is the absolute temperature, *D* is the diffusion coefficient that characterizes the creep process, *G* is the shear modulus, *b* is the Burgers vector, *A* is a dimensionless constant, *d* is the grain size, *s* is the grain size sensitivity, *τ* is the applied shear stress, *n* is the stress exponent, *Q* is the activation energy for the diffusion process that controls the creep behavior, and *D_o_* is the frequency factor for diffusion. Second, the relationship between stress, *τ*, and strain rate, $\dot{\gamma }$, is often sigmoidal [6,7,8,9,10]. Under creep testing conditions, this sigmoidal relationship is manifested by the presence of three regions, as illustrated in Figure 2(a): Region I (the low-stress region), Region II (the intermediate-stress region or the superplastic region), and Region III (the high-stress region). In Region III (the high-stress region), the stress exponent, *n* is higher than 3, the apparent activation energy, and *Q_a_* is higher than that for grain boundary diffusion. Region II (the intermediate-stress region) covers several orders of magnitude of strain rate and is characterized by a stress exponent, *n*, of 1.5 to 2.5, an apparent activation energy, *Q_a_*, that is close to that for boundary diffusion, and a grain size sensitivity, *s* of about 2. In this region, maximum ductility occurs [11,12,13]. Because of this characteristic, Region II is often referred to as the superplastic region. Region I is characterized by a stress exponent of 3 to 5, and an apparent activation energy higher than that for grain boundary diffusion. However, the creep behavior in this region exhibits essentially the same grain size sensitivity noted in Region II. Finally, the percentage contribution of boundary sliding to total strain generally ranges from 50–70% in Region II but it decreases sharply, to approximately 20–30%, in Regions I and III [14,15] as shown in Figure 2(b).

### 2.2. Origin of the Sigmoidal Behavior

The deformation mechanism responsible for Region III is not well established. Early observations have suggested that Region III in superplastic alloys represents normal power-law creep, which controls the behavior of large-grained metals at elevated temperatures [4,5]. These observations [16] included: (a) measurements of high stress exponents in several superplastic alloys, (b) the presence of extensive dislocation activity in the interiors of grains, (c) the occurrence of changes in grain shape (d) increases in the texture after deformation, and (e) the close correspondence between the transition stresses from Region II (the superplastic region) to Region III and those predicted from the equation that describes the dependence of the average subgrain size, λ, formed during the creep of metals on the applied stress [5]. This correspondence implies that Region III occurs at higher stresses where a stable subgrain structure begins to develop [17]. However, the above suggestion that the creep behavior of superplastic alloys in Region III is controlled by the same type of dislocation process, which is dominant in metals at high temperatures, is not entirely satisfactory for two primary reasons. First, experimental data reported for a superplastic copper alloy [10] have revealed an inverse dependence of creep rate on grain size in Region III; this behavior contrasts with that of pure metals at high stresses where creep rates are essentially insensitive to changes in grain size [5]. Second, experimental evidence indicates that at high stresses no well-developed subgrains are formed in the interiors of grains; only dislocations tangles are present [16]. On the basis of the above findings, it seems most likely that Region III is the result of the operation of some form of an intergranular dislocation process, which is influenced by the presence of grain boundaries. It is worth mentioning that there are difficulties in establishing the mechanical characteristics of Region III because of the fast creep rates associated with this region.

As mention earlier, Region II (the superplastic region) is associated with maximum ductility [11,12,13]. The strong sensitivity of steady-state creep rates measured during superplastic flow in Region II to changes in grain size have indicated that boundaries play an important role, which is related to their ability to contribute to deformation through the process of boundary sliding. Over the past four decades, considerable efforts have been made to characterize the nature and significance of such a role in terms of deformation mechanisms. As a result of these efforts, a number of deformation mechanisms were developed or speculated [18,19,20,21,22,23,24]. Depending on the nature of the accommodation process that is necessary to relieve stress concentration, the grain boundary sliding (GBS) models may be divided into two types: diffusional accommodation and dislocation accommodation.

GBS accommodated by diffusion flow is the basis of the model by Ashby and Verrall [18]. This model, which involves a grain-switching event, predicts the presence of a sigmoidal relationship between stress and strain rate, the retention of an equiaxed grain structure, and the absence of a significant dislocation activity. While these predictions are in harmony with experimental evidence, there are several problems associated with the model. These problems were discussed in detail elsewhere [6]. In particular, the model predicts that the apparent activation energies in Regions II and I are the same. This prediction is in conflict with the present finding that Region I is associated with a higher apparent activation energy [6,7,8,9,10].

Several models based on GBS accommodated by dislocation motion were developed [19,20,21,22,23,24]. These models are different in assumptions and details. For example, in the model of Mukherjee [20], large ledges or protrusions on the grain boundary surface provide most the obstruction to boundary sliding. As a result, dislocations are generated at the obstructing ledge. Then, the generated dislocations move into the grain and pileup against the opposite boundary where they climb and are annihilated. On the other hand, the model of Gifkins [21] involves sliding by dislocation movement in the mantle (a narrow region adjacent to boundaries) and accommodation occurs by the glide and climb of dissociated dislocations along boundaries; there is no dislocation activity in the core. Despite various differences in assumptions and details, all models based on GBS accommodated by dislocations can be represented by the following rate-controlling equation that predicts the deformation characteristics reported for the superplastic region, in which ductility exhibits a maximum value:

(2) $$\dot{\gamma }=C\frac{D_{o}Gb}{kT}{\left(\frac{b}{d}\right)}^{2}{\left(\frac{\tau }{G}\right)}^{2}\;exp(\frac{-Q_{gb}}{RT})$$

where *C* is a constant and all other terms have been defined previously; the values of C for the above mentioned models are given in Table 1.

**Table 1:** Proposed deformation mechanisms for the superplastic region (Region II).

Model	C	Comments
Ball-Hutchison [19]	600	Sliding of group of grains; dislocations are created at triple points and annihilated by the process of climb into opposite grain boundaries D = D_gb_
Mukherjee [20]	12	Grains slide individually; dislocations are produced by ledges and protrusions D = D_gb_
Gifkins [21]	384	Dislocation movement by glide and climb in the mantle along the adjacent grains D = D_gb_
Gittus [22]	320	Pile up of boundary dislocations at interphase boundaries D = D_IPB_, *τ_o_* is ignored for Region II
Arieli-Mukherjee [23]	480	The creation of dislocations on a solute-free mantle by Bardeen-Herring multiplication D = D_gb_
Paidar-Takeuchi [24]	30	Grain rolling; GBS by the glide of GB dislocations on sliding grain facets; accommodation by the climb of GBS dislocations on facets with normal stresses D = D_gb_ Ω = atomic size = 0.7b^3^ δ = boundary thickness = 2b

Early explanations for the origin of Region I were centered around: (a) the operation of temperature-insensitive threshed stress processes [25], (b) the emergence of new deformation mechanism [6,7], or (c) the occurrence of concurrent grain growth [26]. However, as concluded elsewhere [27,28], these explanations are not entirely consistent with available experimental evidence. 

**Figure 3:**
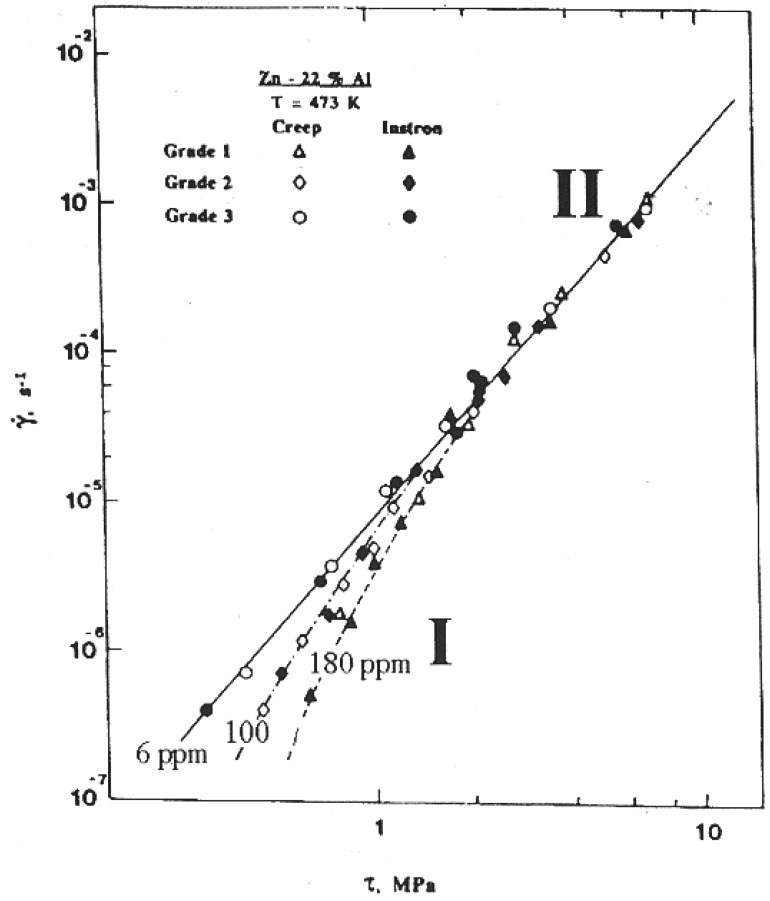
Shear strain rate *vs.* shear stress (logarithmic scale) for three grades of Zn-22% Al having a grain size of 2.5 µm at 473 K.

**Figure 4:**
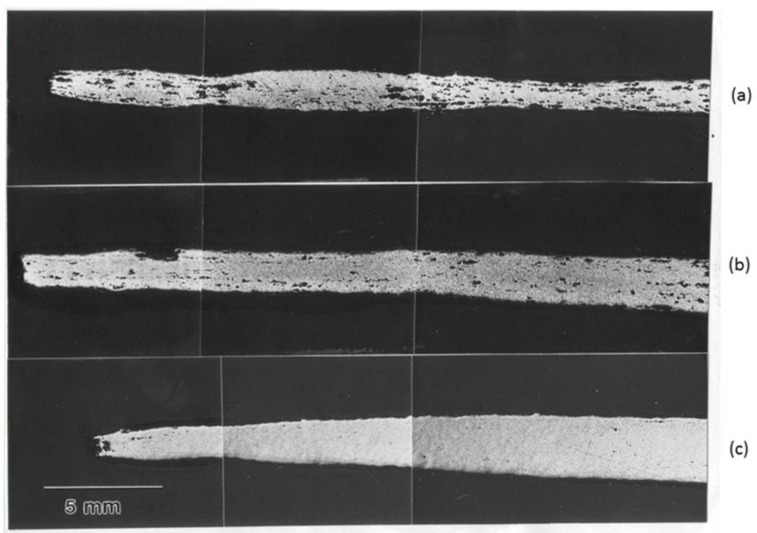
Cavitation in Zn-22% Al grades at a strain rate of 1.33 × 10^−5^ s^−1^. (**a**) grade 1 (180 ppm of impurities); (**b**) grade 2 (100 ppm of impurities); and (**c**) grade 3 (6 ppm of impurities).

**Figure 5:**
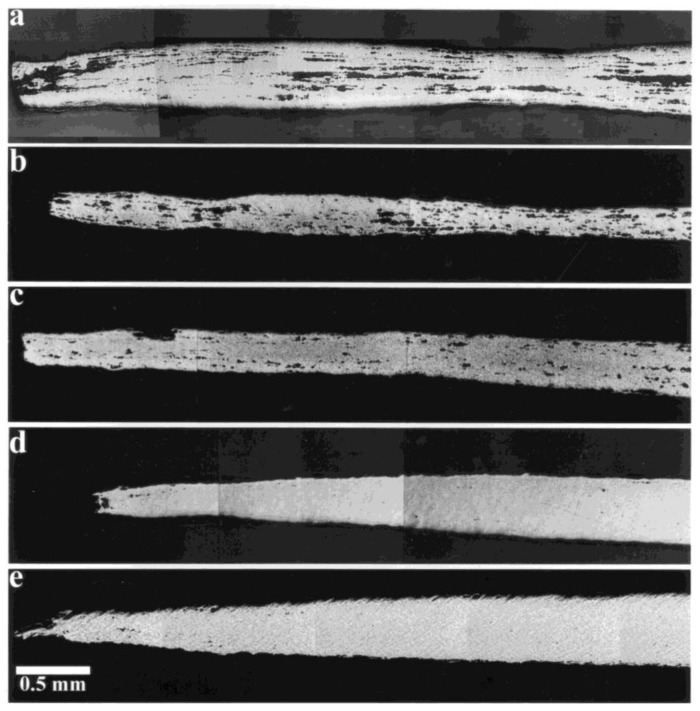
Cavitation in different grades of Zn-22% Al tested at 473 K and a strain rate of 1.33 × 10^−5^. (**a**) Zn-22% Al-014 % Fe; (**b**) grade 1 with 180 ppm impurities; (**c**) grade 2 with 100 ppm impurities; (**d**) high-purity Zn-22% Al; and (**e**) Zn-22% 22% Al-0.13% Cu.

It has been suggested [27] on the basis of an analysis of superplastic flow at low stresses, that Region I behavior may be a consequence of the operation of a threshold stress process whose origin is related to the segregation of impurity atoms at boundaries and their interaction with boundary dislocations; in this case, the threshold stress, *τ_o_*, is equivalent to the stress that must be exceeded before boundary dislocations can break away from the impurity atmosphere and produce deformation. Consistent with the above suggestion are several observations. First, it was demonstrated that Region I behavior was influenced by the purity level of the alloy [28,29,30,31]. This finding was reflected in three primary observations: (a) Zn-22% Al did not exhibit as indicated by Figure 3, Figure 4 and Figure 5 Region I when the level of impurities in both alloys was reduced to about 6 ppm (throughout this work ppm will refer to wt. ppm, unless otherwise stated) [28], (b) increasing the impurity level at constant initial strain rate reduced ductility [32]. Second, creep data reported for several grades of Zn-22% Al containing different levels of impurities [28], in particular Fe [30], revealed the presence of a threshold stress whose characteristics were consistent with various phenomena associated with boundary segregation. For example, the temperature dependence of the threshold stress was described by $\tau_{o}/G=\beta_{o}\;exp(Q_{o}/RT)$ is similar in form to $c=c_{o}\;exp(W/RT)$ (*c_o_* is the average concentration of impurity, and *W* is the interaction energy between a boundary and a solute atom), that gives, to a first approximation, the concentration of impurity atoms segregated to boundaries, *c*, as a function of temperature [33]. Third, the presence of other impurities in addition to Fe in Zn-22% Al resulted in enhancing cavitation [32]. This observation appears to be consistent with the synergistic effects associated with impurity segregation at boundaries [34,35]. Fourth, the Fe level (120 ppm) at which the threshold stress for creep in Zn-22% Al appears to approach a limiting value most likely represents the concentration at which boundary sites available for Fe segregation approach a saturation limit [30]. Fifth, experimental results on cavitation revealed the following observations: (a) cavities were not observed in high-purity Zn-22% Al [31,32], and (b) the extent of cavitation in Zn-22% Al was dependent on the impurity content of the alloy [31]. Findings (a) and (b) are illustrated in Figure 4. The observed correlation between the level of impurities and the extent of cavitation in Zn-22% Al is most probably related to effects associated with the presence of excessive impurities at boundaries due to their segregation [32,36,37]. Finally, a detailed investigation [38] was conducted to study the effect of Cu, as a selected impurity, on superplastic deformation and cavitation in Zn-22% Al. The results have shown that cavitation is not extensive in Zn-22% Al doped with 1300 ppm of Cu (Figure 4). This characteristic is essentially similar to that reported for high-purity Zn-22% Al [31] but is different from that documented for a grade of the alloy containing a comparable atomic concentration of Fe [32] (Figure 5). This observation appears to be consistent with the expectation that impurities vary greatly in tendency to segregate at boundaries. Also, this observation is in harmony with the absence of Region I in the logarithmic plot of strain rate against stress for Zn-22% Al doped with Cu [38].

**Figure 6:**
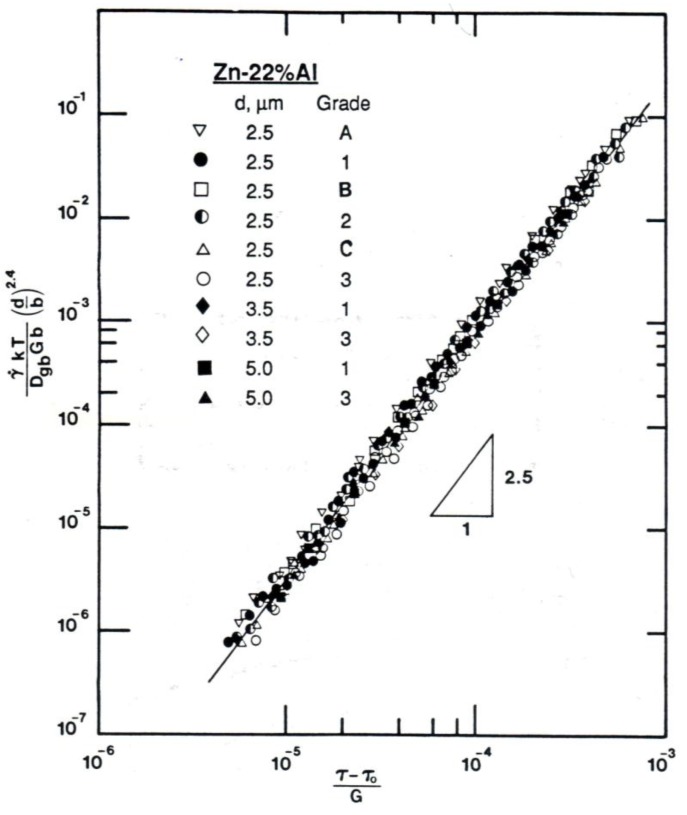
A plot of normalized creep rate *vs.* normalized effective stress for several grades of Zn-22% Al containing different impurity levels. Data were taken from [30].

The concept of an impurity-dominated threshold stress signifies that the same deformation process controls both Region II (the superplastic region) and Region I (the low-stress region) and that the apparent difference in stress and temperature dependencies between the two regions are the result of the increasing importance of *τ_o_* with decreasing creep stress in grades containing sufficient impurity levels. Consistent with this concept is the finding that the experimental data obtained for superplastic alloys in Regions I and II at various temperatures can be described by a single deformation process that incorporates, *τ_o_*, and may be given by [27,28,30]:

(3) $$\frac{\dot{\gamma }kT}{D_{gb}Gb}=B{\left(\frac{b}{d}\right)}^{s}{\left(\frac{\tau -\tau_{o}}{G}\right)}^{n}$$

where *s* is about 2 and *n* is about 2.5. Such a description is illustrated in Figure 6, where the normalized creep rate multiplied by the normalized grain size s plotted as a function of the normalized effective stress. It is clear from the plot in Figure 4 that the data on Zn-22% Al, regardless of the value of the grain size and the level of impurities, coalesce into a straight line, whose slope is about 2.5 (*n* = 2.5) and which extend over more than five orders of magnitude of strain rate. These characteristics demonstrate that the following important point: superplasticity does not represent a transition region between the domains of two deformation processes but arises from a single deformation mechanism. 

### 2.3. Effect of Impurities on Boundary Sliding

**Figure 7:**
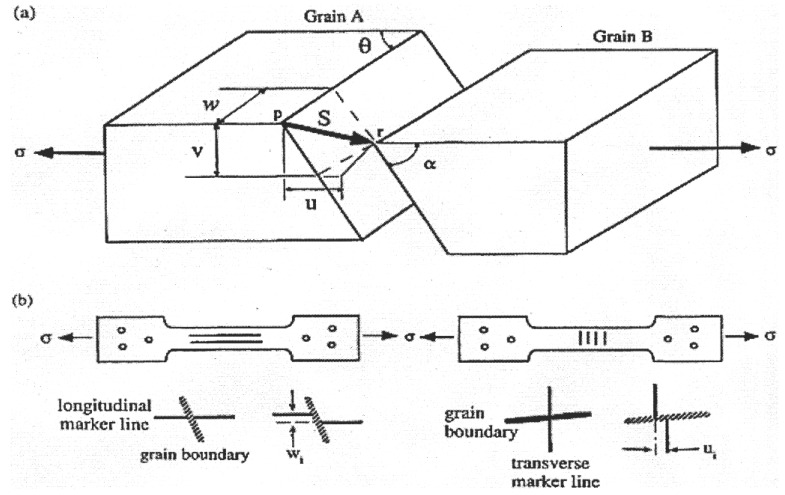
(**a**) Schematic representation of grain boundary sliding; (**b**) Schematic configuration of marker lines, longitudinal and transverse and their offsets.

Grain boundary sliding (GBS) is a process that occurs during the high temperature creep of polycrystalline materials and in which one grain slides over another grain under the action of a shear stress. A schematic representation of sliding is shown in Figure 7(a). As a result of the sliding of the two grains, offsets are produced at their common boundary. In the figure, *pr* is the sliding vector, *u* is the component of sliding resolved along the stress axis, *v* is the component measured perpendicular to both the stress axis and the specimen surface, and *w* is the component measured perpendicular to the stress axis but in the plane of the surface. Also, as shown in Figure 7(a), the orientation of the grain boundary is defined by two angles *θ* and *α*: *θ* is the angle between the stress axis and the trace of the boundary in the plane of the surface, and *α* is the internal angle made by the boundary trace on a longitudinal section cut perpendicular to the surface.

The occurrence of micrograin superplasticity in metallic systems requires a stable and equiaxed grain size of less than 10 µm. This requirement along with the strong sensitivity of steady-state creep rates measured during superplastic flow to changes in grain size, *d*, has demonstrated the significant influence of boundaries on the superplastic behavior. Over the past four decades, considerable efforts have been made to characterize the nature and significance their role. For example, as mentioned previously, the concept of boundary sliding accommodated by some form of dislocation activity was adopted in developing several deformation models [19,20,21,22,23,24] that treated steady-state superplastic deformation in Region II. In addition, many investigations have been performed in tension on several different superplastic alloys to evaluate the significance of GBS in the above three regions of behavior. In these investigations, measurements of sliding offsets along prescribed marker lines on the surface of tensile specimens were taken and the contribution of GBS to the total strain was estimated using appropriate equations. In particular, in superplasticity experiments, the longitudinal offset, *u*, and/or the transverse offsets *w*, are considered in calculating the strain contribution from boundary sliding to total strain. As described elsewhere [14,15], the strain due to boundary sliding, *ξ* , is obtained from $\bar{u}$ and $\bar{w}$ through the following expressions:

(4a) $$\varepsilon_{gbs}=\psi \bar{u}/\bar{L}$$

(4b) $$\varepsilon_{gbs}=\phi \bar{w}/\bar{L}$$

where *ψ* is a geometric constant which is equal to 0.8 for the longitudinal offset, $\bar{u}$ is the average offset. $\bar{L}$ is the average linear intercept grain size, *f* is a geometric constant that is equal to 1.5 for the transverse offset [14,15], and $\bar{w}$ is the average transverse offset. The method of calculating *ξ* from *u* is applicable only when the grains are equiaxed, a condition that is satisfied during superplastic deformation. The contribution of boundary sliding to the total strain, *x*, is calculated from the equation:

(5) $$\xi =\varepsilon_{gbs}/\varepsilon_{t}$$

In taking measurements of GBS, several steps are carried out. First, prior to testing, one of the flat surfaces of each specimen is polished to a mirror-like scratch-free surface, and very fine lines (marker lines), either parallel or perpendicular to the specimen axis, are placed on the polished gauge surface. One method of creating such lines is by drawing a lens tissue containing 1 mm diamond paste across the surface only once in either the longitudinal or the transverse direction. Longitudinal marker lines are used to measure the sliding offset, *w*, perpendicular to the stress axis. Transverse marker lines are used to measure the sliding offset, *u*, parallel to the stress axis. Figure 5(b) illustrates the two types of marker line, longitudinal and transverse, and the two classes of offset, *w* and *u*. It is also possible to take measurements of sliding using a printed-grid technique. However, as reported elsewhere [39], the technique can be unsatisfactory due to resolution difficulties in the scanning electron microscope. Second, tensile specimens are deformed at a constant temperature on a testing machine operated at a constant cross-head speed. Tensile tests are conducted to a predetermined strain in the range of 20%–100% at various initial strain rates. Third, after testing and cleaning in an ultrasonic cleaner, the specimens are examined in the scanning electron microscope (SEM) and a number of representative photo-micrographs are taken within the gauge length. These photo-micrographs are enlarged and individual sliding offsets are measured. In general, about 200–500 readings are taken on each one of the specimens.

Superplastic alloys such as Zn-22%Al and Pb-62%Sn have two phases, and in this case, there are two intercrystalline boundaries (for example: Al-Al and Zn-Zn) and an interphase boundary (for example: Al-Zn). In order to ensure that sampling of measurement for GBS is not in favor of a certain type of boundary, the number of each type of boundary counted experimentally were selected in some investigations according to an approach that Gifkins proposed [40]. The approach by Gifkins [40] is based on the following assumptions: (a) the ratio of the volume fraction of the two phases of a micrograin superplastic alloy is equivalent to the ratio in the linear transverse intercept, and (b) grains make a single transverse array. If the ratio of the volume fraction of *α* and *β* phases in the alloy is *f*, then this ratio, on the basis of the above assumptions, may be expressed as:

(6a) $$X{\bar{L}}_{\alpha }/Y{\bar{L}}_{\beta }=f$$

where *X* and *Y* are the number of *α* and *β* grains, respectively, and ${\bar{L}}_{\alpha }$ and ${\bar{L}}_{\beta }$ are the linear sizes of *α* and *β* phases, respectively. The above expression may be rewritten in a second form using the following consideration. If *x (α-α)* boundaries and *y (α-β)* boundaries are counted, there would be *x (α)* grains forming *(α-α)* boundaries and *y/2* *(α)* grains forming *α-β* boundaries, *i.e*., *X* = *x* + *y/2*. Then, the number of *β* grains, *Y*, and the number of *(β-β)* boundaries, *z*, are given, respectively, by

(6b) $$Y=\frac{1}{f}\left(\frac{{\bar{L}}_{\alpha }}{{\bar{L}}_{\beta }}\right)\left(x+\frac{y}{2}\right)$$

and

(6c) z=Y−y/2

Despite minor differences in the procedures and equations used in GBS estimates, the results reported in the investigations [14,15,39,40] are in general consistent and show that, at low elongations (20–30%), the percentage contribution of GBS to total strain generally ranges from 50–70% in Region II but it decreases sharply to approximately 20–30% in Regions I and III. It should be mentioned that GBS measurements in ultrafine-grained Zn-22% Al yielded [41] *ξ* = 44%–50% when *n*~2.

The value of *ξ* in Region II (~60%) suggests that there is a missing strain of about 40%. This missing strain is too large to be explained in terms of diffusional creep and/or dislocation motion considering two well-documented observations related to superplastic deformation: the contribution of diffusional creep to the total strain is not significant [42] and strain produced by lattice dislocations is negligible [43]. Langdon [44] has argued that there is no missing strain and that boundary sliding and the associated accommodation process account for essentially all strain produced during superplastic flow in Region II. His argument [44] was based on an analysis of the process of measuring sliding using marker lines parallel to the tensile axis in a two dimensional array of hexagonal grains. The analysis [44] has led to the prediction that *ξ* exhibits a minimum value of 45% when sliding is not accommodated and assumes a maximum value of 90% under the condition that sliding is fully accommodated. On the basis of this prediction, Langdon [44] has concluded that since the accommodation of sliding is not fully required at the surface of a tensile specimen, the experimental values of *ξ* obtained from surface marker lines are expected to be close to the lower bound of the range of 45%–90%.

Grain boundary measurements in three grades of Zn-22% Al containing different impurity levels indicated that three grades of the alloy exhibited essentially the same value for *ξ* in Region II [45]. The measurements along with those reported for Pb-Sn [46] signify that impurity level has no noticeable effect on steady-state creep behavior in this region. For illustration, see Figure 6(a) and Figure 6(b).

**Figure 8:**
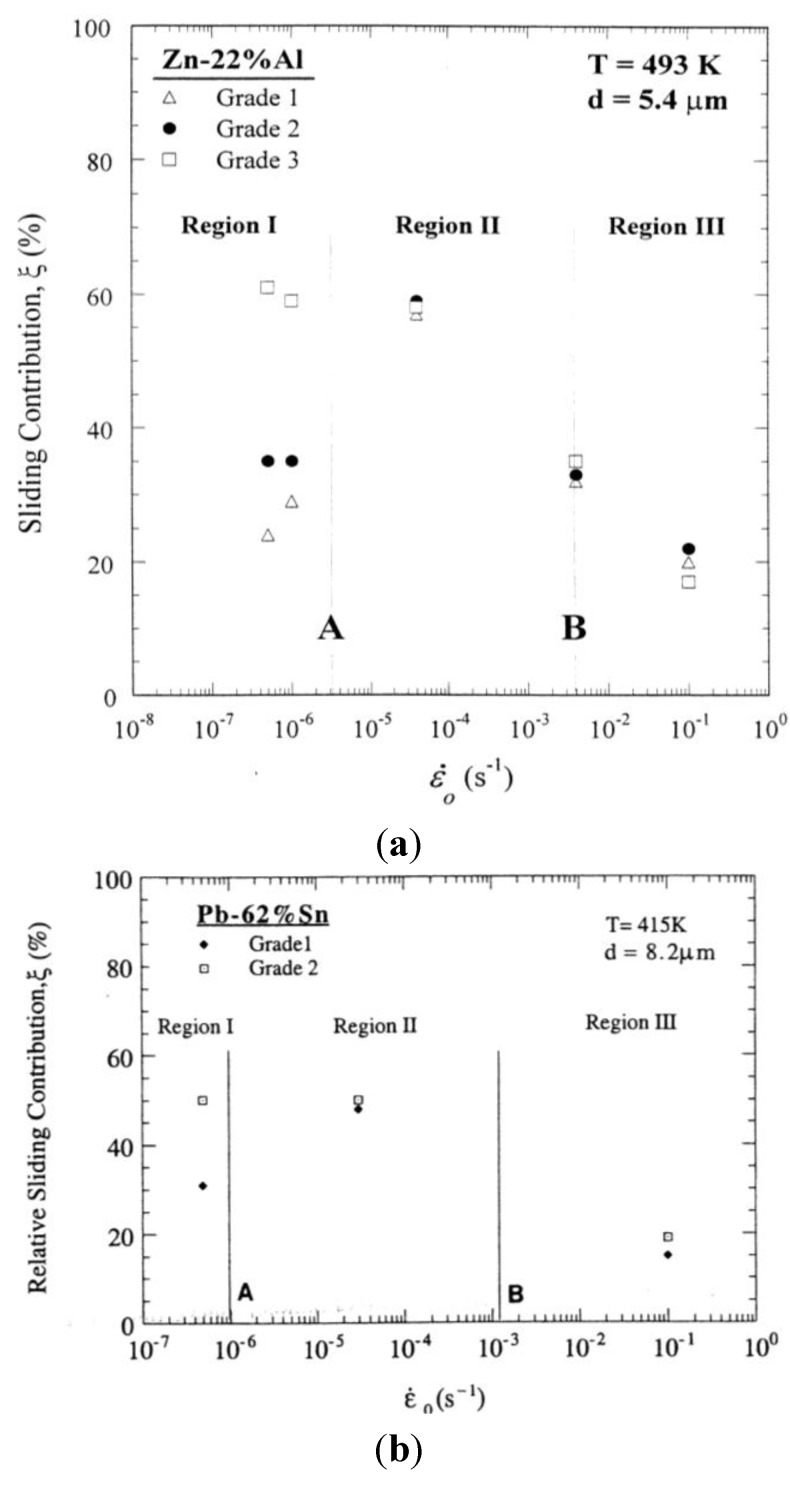
(**a**) The contribution of boundary sliding to the total strain as a function of strain rates for grades 1, 2, and 3 of Zn-22%Al containing 180, 100, and 6 ppm of impurities, respectively; (**b**) The contribution of boundary sliding to the total strain as a function of strain rates for grades 1and 2 of Pb-62% Sn. The vertical lines A and B represent the transitions from Region I to Region II and from Region II to Region III, respectively.

As stated earlier, experimental results on Zn-22%Al and Pb-62 Al indicated that Region II and I are controlled by the same deformation process and that the apparent differences in the deformation characteristics between the regions is a reflection of the presence of an impurity–dominated threshold stress, which arise from boundary segregation. Accordingly, in the absence of boundary segregation, it is expected that boundary sliding behavior at low strain rates would be similar to that at intermediate strain rates, where the superplastic region dominates. In this case, there would be no significant difference between the two ranges of strain rates in terms of the contribution of sliding to the total strain. The results reported for sliding characteristics in high-purity Zn-22% Al (6 ppm of impurities) [45] and high-purity Pb-62% Sn (5 ppm of impurities) [46] has verified such an expectation as shown by Figure 8(a) and Figure 8(b), respectively. For example, it was found that in the former alloy, *ξ* is about 60% at both intermediate and low strain rates. The high value of *ξ* in high-purity Zn-22% Al at low strain rate is consistent with two experimental observations related to the alloy [28,31]: (a) Region II at intermediate strain rates extends to low strain rates with no evidence for Region I behavior, and (b) cavitation at low strain rates, like that at intermediate strain rates, is not extensive.

On the other hand, the occurrence of boundary segregation is expected to reduce the contribution of sliding at low strain rates where Region I is normally observed. This expectation is based on the argument [30] that the presence of impurities may influence accommodation processes for boundary sliding, which in general include boundary migration, diffusional flow, dislocation motion, or cavitation. For example, the presence of excessive impurities at boundaries may affect boundary migration in two ways. First, impurities may produce a strong dragging effect on migrating boundaries. As result, grain boundary migration (GBM), which is a fast process at intermediate stresses (Region II) [47], may become too slow to fully accommodate boundary sliding in this range of stresses, leading to a decrease in the amount of sliding and an increase in the extent of cavitation. Second, the presence of excessive impurities at boundaries may lead to the formation of precipitates. These precipitates may reduce sliding rate by changing originally straight boundaries to serrated ones [33]. Such a change in boundary configuration could be the result of the following two processes: the pinning of a boundary at various points by precipitate particles and the occurrence of limited GBM (due to impurity drag at low stresses).

### 2.4. Cavity Stringers

**Figure 9:**
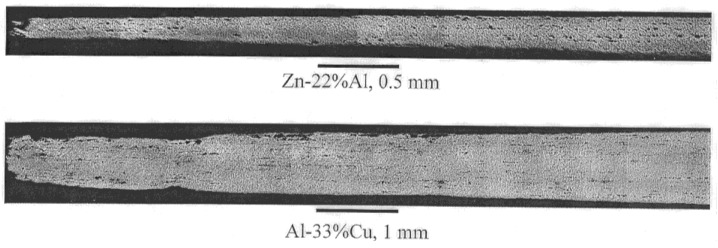
Cavity stringers in superplastic alloys.

It is well established that during superplastic deformation, most materials develop cavities, which grow and coalesce, leading to cavitation damage. Such damage in turn gives rise to premature failure of the material, thereby limiting the use of superplastically formed components. A major characteristic of cavitation in superplastic alloys is that cavities usually display an aligned configuration [47,48,49,50,51,52,53] as demonstrated by Figure 9; a group of cavities aligned in a specific direction is referred to as a cavity stringer. The morphology of cavity stringers varies from one material to another and is controlled by the general variables of superplastic cavitation such as strain, strain rate, and temperature. Interaction between cavity stringers also influences the final configuration. 

During prior thermomechanical treatment, most superplastic materials are rolled either to produce the fine grain size necessary for superplastic deformation or as part of the processing procedure. Large particles, if present, can therefore break down to align as small particles along the rolling direction. Since superplastic materials have commonly been tested with the tensile axis parallel to the rolling direction, it was suggested that during testing, cavity nucleation at these particles leads to the formation of cavity stringers [49,52]. This suggestion was verified by the observation that changing the orientation of the test samples led to a corresponding change in the arrangement of cavity stringers [49,52]. Following these results, however, the effect of rolling direction on other quasi-single phase and microduplex alloys was studied and it was found that cavity stringers always align parallel to the tensile axis, regardless of the rolling direction [53,54].

**Figure 10:**
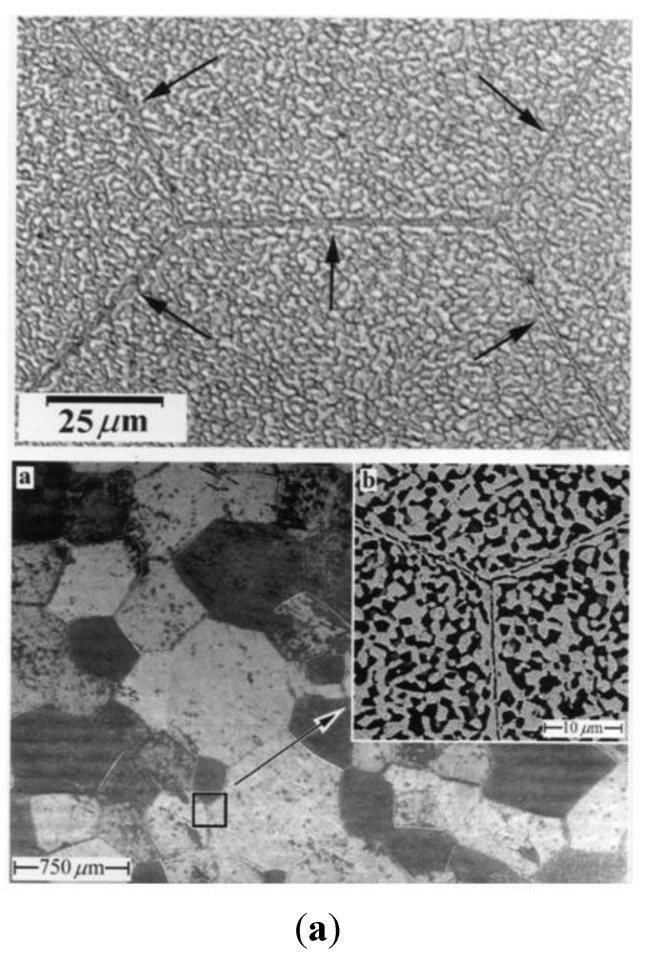

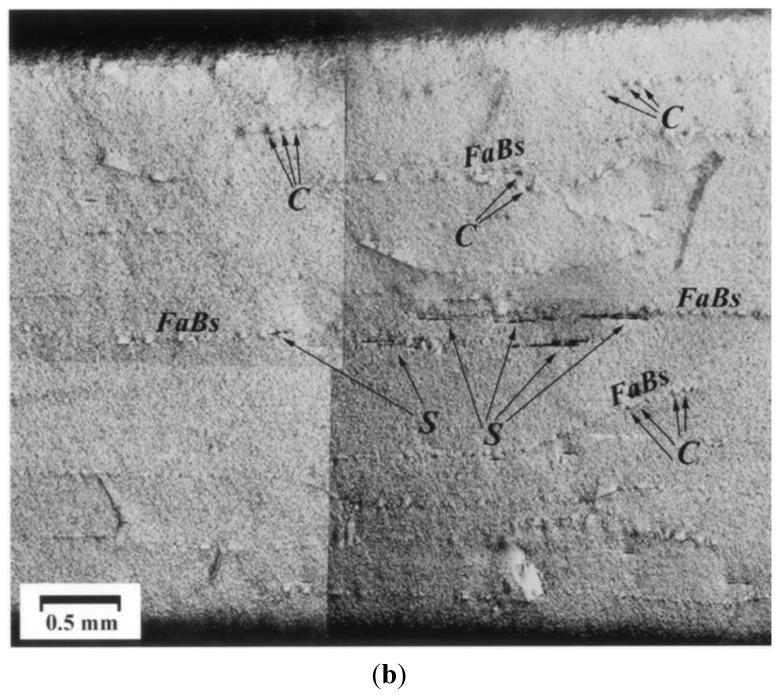
(**a**) FαBs in Zn-22% Al; (**b**) Formation of cavities and stringers on *FαBs*. Tensile axis is horizontal.

The key to uncovering the mystery about the origin of cavity stringers was provided by two pieces of information. First, experimental results reported for Zn-22 wt% Al have shown that the extent of cavitation increases with increasing impurity content [31] (see Figure 4). Second, the results of a study [55] on the effect of heat treatment on the microstructural behavior of Zn-22 wt% Al have revealed that groups of fine *α* (Al-rich) and *β* (Zn-rich) phases, which form by spontaneous decomposition, are encompassed by former *α* boundaries (*FαBs*) that consist of fine elongated *a* grains; *α* boundaries (*FαBs*) divide the microstructure into equiaxed domains containing fine grains of *α* and *β* phases. Figure 10(a) shows this microstrural feature. In addition, microstructural observations following deformation have indicated [55] that these groups of *α* and *β* phases behave as independent domains and that *FαBs* tend to align parallel to the tensile axis during superplastic deformation. These findings were significant in two ways: (a) they suggested that the presence of *FαBs* could be used as a tracer to monitor various activities which accompany superplastic flow, and (b) they implied that a link might exist between the occurrence of cavity stringers and the presence of *FαBs*.

Detailed investigations [56] that involved monitoring both the evolution of *FαBs* and the development of stringers led to the following new information:

- *FαBs* serve as favorable cavity nucleation sites as demonstrated by Figure 8(b). This role is attributed in part to the shape of the grains in *FαBs* (elongated) and in part to impurity segregation at these boundaries [56].
- There is a direct correspondence between the evolution of these two substructural features: *the nucleation of cavities on FαBs and the formation of cavity stringers*

**Figure 11:**
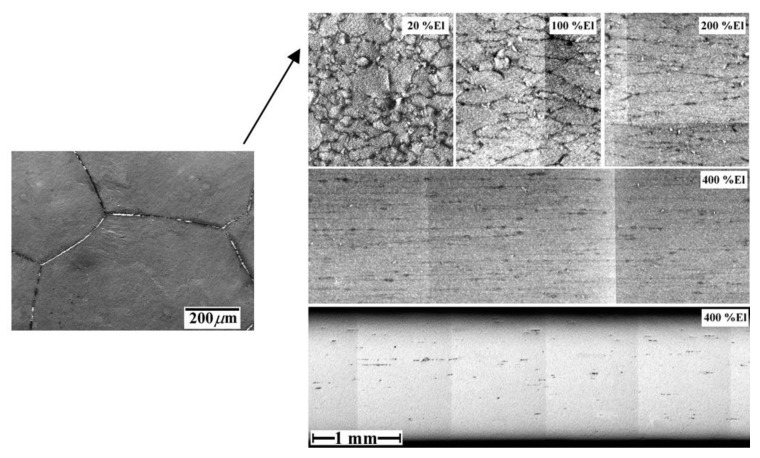
Correlation between the rotation of *FαBs* during superplastic flow and the formation of cavity stringers.

On the basis of the above information, the following mechanism was proposed [57] to account for cavity stringers in Zn-22 wt%Al. Upon deformation, cavities begin to nucleate at *FαBs*. As deformation continues, these boundaries change their orientation, approaching the tensile axis and resulting in cavity stringers aligned in this direction. This process is illustrated in Figure 11.

The presence of *FαBs* in Zn-22 wt%Al and their initial orientation with respect to the tensile axis lead to the formation of cavities along inclined directions that, upon superplastic deformation, tend to align with the tensile axis. Since *FαBs* are not a common substructural feature in other superplastic alloys that also exhibit cavity stringers, a general mechanism was proposed [57] to explain how the formation of cavity stringers is, in general, accommodated by superplastic flow. 

**Figure 12:**
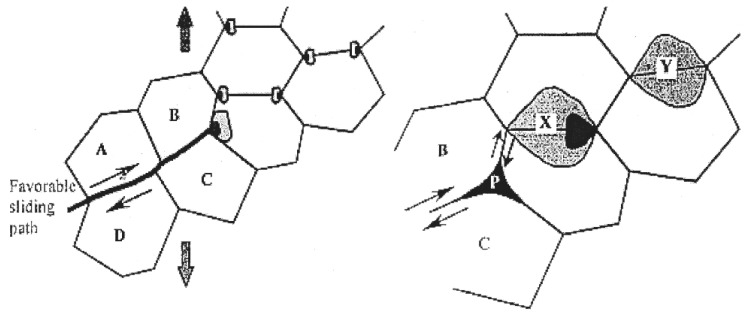
General model for the formation of cavity stringers.

By monitoring the behavior of *FαBs* during superplastic deformation, it was suggested [57] that, in general, the formation of cavity stringers is essentially accommodated by superplastic flow. This suggestion represented the basis of a mechanism that provides a possible explanation for the formation of cavity of stringers under micrograin superplasticity conditions. The mechanism involves two main steps: (a) the formation of rows of cavities at directions inclined to the tensile axis, and (b) as a result of superplastic flow, these rows change their orientation, and approach the tensile axis in a fashion similar to that of *FαBs*. This process is illustrated in the following scheme. It is assumed that during a burst of boundary sliding a group of grains (labeled *A*, *B*, *C*, and *D* in Figure 12) slide as a unit, until blocked by unfavorably oriented grain boundaries. This generates a stress concentration at the corresponding triple junctions shown in Figure 12. In the absence of accommodation by diffusion, deformation, and/or boundary migration, the local stress concentration is relieved by the opening of a cavity at point *P*. According to the results of numerical calculations on boundary sliding and cavitation [58], the opening of such a cavity results in stressing facets *X* and *Y* to a higher level than other transverse facets. As a result, further cavity nucleation would be favored at these transverse boundaries. This process, which continues until the accommodation is damped out, could lead to the formation of a short row of cavities, which is inclined to the tensile axis. Following the formation of these short rows of cavities, flow accommodated alignment, as reflected in the behavior of *FαBs*, would take place to ultimately form cavity stringers parallel to the tensile axis. In the preceding discussion, it is suggested that the alignment of cavities in the form of cavity stringers is naturally promoted by superplastic flow. On the basis of this suggestion, it is expected that cavity stringers would form along the tensile axis of superplastic materials as long as the following conditions exist. First, stress concentrations resulting from sliding of an individual grain or a group of grains are relieved by opening cavities, *i.e.*, sliding is not accommodated by diffusion, deformation and/or boundary migration. Second, early failure due to the interlinkage of cavities in a direction transverse to the tensile axis or due to the development of a sharp neck does not occur, permitting cavities to experience flow accommodated alignment. Finally, cavity distribution is nonuniform and very extensive cavitation does not occur, since under these circumstances, even if the material shows high elongations before failure, the directionality of the cavities will be obscured.

As mentioned earlier, large particles, if present in a superplastic alloy, can break down during rolling to align as small particles along the rolling direction. Under this condition, particle stringers pre-exist parallel to the tensile axis of a superplastic material due to aligned nucleation, cavity stringers will form in the same direction. This occurs in the early stages of deformation, and the role of flow-accommodated alignment becomes overshadowed during subsequent deformation. Cases in which cavity stringers initially develop perpendicular to the tensile axis have been reported to fail at low strains, before the stringers are given the opportunity to align parallel to the tensile axis as described earlier. In addition, it is expected that a situation may arise in which the rate of transverse interlinkage of individual cavities is comparable to that of superplastic flow assisted alignment, leading to an intermediate case where, due to premature failure, rows of cavities are not able to completely align parallel to the tensile axis. This expectation is consistent with the observation made in some materials, which show stringers that are inclined to the tensile axis [59]. Other prevalent situations where cavity stringers are aligned parallel to the tensile axis regardless of the rolling direction clearly occur as a consequence of superplastic flow

### 2.5. Dislocation Activity during Superplastic Deformation

As mentioned above, the models of GBS accommodated by dislocation motion incorporate lattice dislocation activity as a major component in the rate-controlling process of superplastic flow. Over the past two decades, several approaches were adopted to monitor lattice dislocations during superplastic deformation. Melton *et al.* [60] reported indirect evidence of dislocation motion by texture measurements performed on a superplastically deformed material. Samuelsson *et al.* [61] used small precipitates as internal pinning points for dislocations in Zn-40wt%Al and observed dislocation processes in a strain rate region that spanned the optimum strain rate for superplasticity in the material. Valiev and Langdon [43] measured lattice dislocation strain from the changes in separation between marker lines contained within a single grain of deforming Pb-62wt%Sn eutectic alloy and obtained indirect evidence for the movement of some lattice dislocations. On the other hand, Nicholson [62] found no dislocations by using precipitates as obstacles to dislocation motion. Also, Naziri *et al.* [63] reported no dislocation movement *in situ* studies of superplastic deformation of the Zn-22%Al eutectoid in a high voltage transmission electron microscope (TEM). 

**Figure 13:**
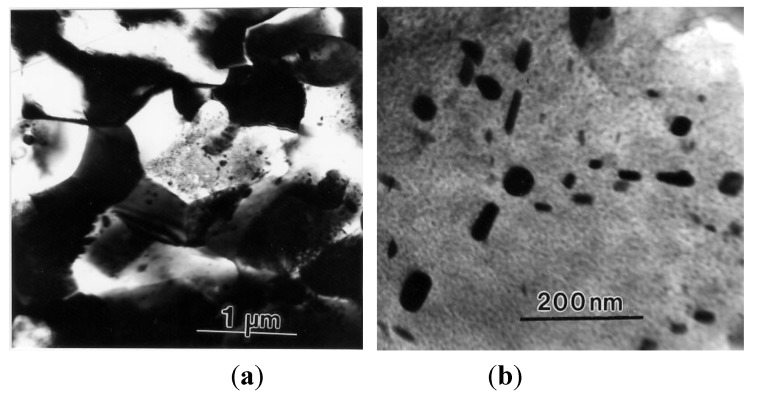
TEM micrographs of the starting material: (**a**) low magnification showing a group of grains; and (**b**) high magnifications microscopy showing dispersion distribution in a grain.

**Figure 14:**
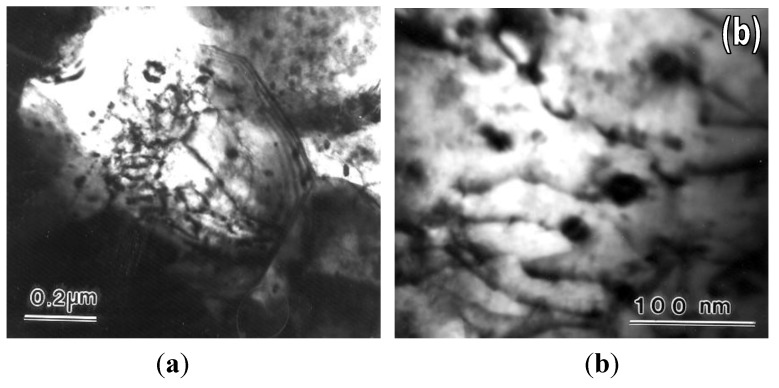
Transmission electronic microscopy of dispersion-bearing Zn-22% Al crept in Region I (*τ* = 2.5 MPa and T = 463 K). (**a**) Low magnification microscopy showing a group of grains with the central grain containing dislocations; (**b**) High magnification weak beam microscopy showing dislocations attached to dispersion particles in the interior of a grain.

The preceding discussion showed that evidence regarding lattice dislocation activity during superplastic flow was not entirely satisfactory and that efforts were needed to obtain definitive information on the role of lattice dislocation during micrograin superplasticity. Xun and Mohamed [64,65] provided such information that was obtained from experiments on the superplastic Zn-22%Al eutectoid, which contained nanometer-scale dispersion particles. These particles were introduced in the matrix of the alloy via powder metallurgy followed by cryomilling [66]. Figure 13 shows the distribution of these nano-particles. Transmission electron microscopy observations made on specimens crept at a strain rate near the center of the superplastic region have revealed that the initial microstructure is dislocation free; that after deformation, only some grains contain dislocations, which interact with dispersion particle; and that the configurations of the lattice dislocations in the interiors of these grains are suggestive of viscous glide and single slip. The above characteristics, which are illustrated in Figure 14, are consistent with the model of Ball and Hutchison [19] that during a burst of boundary sliding, a group of grains slide as a unit until blocked by an unfavorably oriented grain; a triple point is present. This process produces stress concentration at the triple point. The local stress concentration may be relieved by the generation and movement of lattice dislocations in the blocking grain. When the interiors of the blocking grain is free of obstacles, dislocations move and then pile up at the opposite grain boundary until their back stress prevents further generation of dislocations. The dislocations at the head of the pile-up climb into and along the grain boundary [67], and the continual replacement of the dislocations would permit further grain boundary sliding.

### 2.6. Utilization of the Phenomenon of Micrograin Superplasticity

Micrograin superplasticity is characterized by large elongations that are equal to or greater than 500%. However, these large elongations are usually attained at strain rates in the range of 10^−5^–10^−3^ s^−1^. Such a strain rate range is slow for commercial forming of structural materials. However, recent advances in superplasticity have led to a new field of high-strain rate (HSR) superplasticity, which is very beneficial for commercial applications. The basic idea behind this new field is the finding [68] that ductility-strain rate curve of a micrograined superplastic alloy shifts to higher strain rates with decreasing the grain size. This finding suggests an approach to induce high-strain rate (HSR) superplasticity in commercial alloys: HSR superplasticity can be produced via grain refinement. Equal-Channel Angular pressing (ECAP) [69] and cryomilling followed by consolidation [66] are two processes that can be used for the purpose of grain refinement. Mohamed *et al.* [70,71] adopted the latter technique. 

Cryomilling, which is a variation of conventional mechanical milling, involves milling a metallic powder in liquid nitrogen or liquid argon at cryogenic temperatures [66]. Available information [66] has shown that cryomilling is much more effective than milling at room temperature with a similar energy level. In general, milling or cryomilling produces nanostructures by the structural decomposition of large-grained structures as the result of severe cyclic deformation in the powder. The technique has several advantages. First, it is simple and cost effective, requiring relatively inexpensive equipment (on the laboratory scale). Second, it is applicable to essentially all classes of metals, with the possibility of scaling up to production of tonnage quantities. Third, it can produce bulk near nano grained or ultrafine-grained (UFG) materials that are nearly free of porosity.

The alloy selected for refinement is 5803 Al. The composition of the alloy is: Al-94.7%, Mg-4.86%, and Cr-0.7 %. According to available information, the alloy exhibits elongation of 15% and 40% at room temperature and 400, 25, 26 K, respectively. The alloy is being used in several applications including transportation vehicles missile components, welded pressure vessels, and parts of oilrigs. 

**Figure 15:**
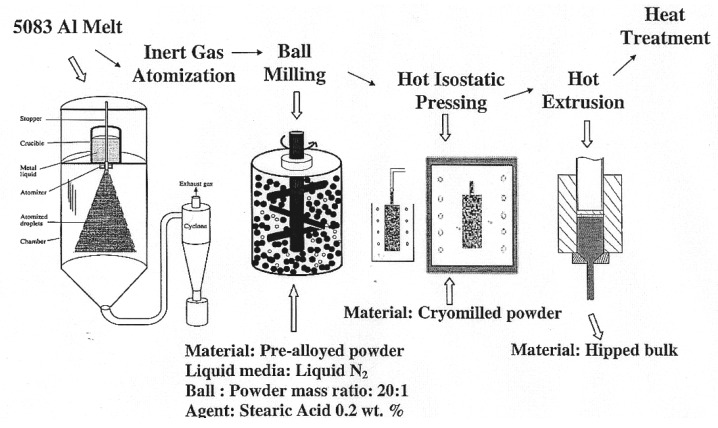
Cryomilling followed by consolidation and extrusion.

The target material, 5083 Al was first spray atomized. The spray atomized powder with a particle size less than 150 µm was mechanically milled using stainless steel balls in liquid nitrogen slurry (cryomilling). The milled powders were packed in aluminum cans in an inert atmosphere before hot degassing under vacuum. Consolidation of the canned powders was completed by HIPping (hot isostatic pressing). Finally, HIPped billets were extruded to remove any remaining porosity and to enhance mechanical properties. Figure 15 summaries the steps. 

**Figure 16:**
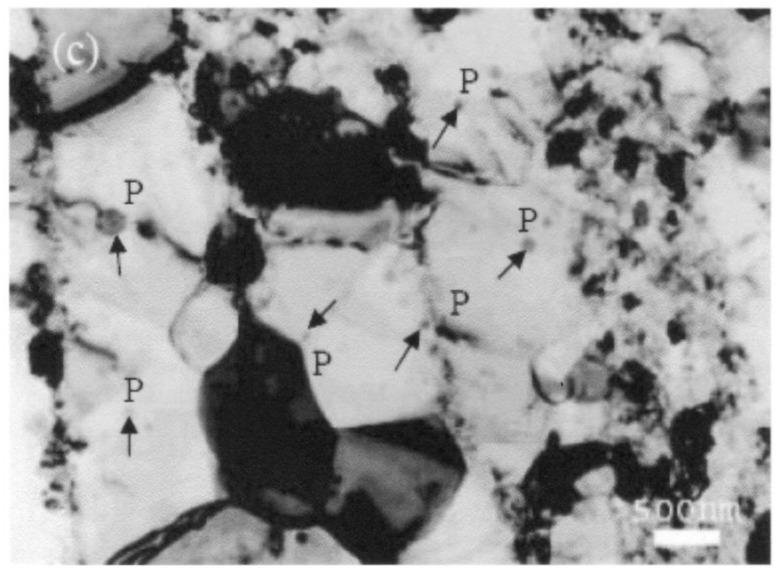
High magnification TEM micrograph of an annealed sample at 573 K for 50 h showing the presence of nanoscale dispersion particles (marked “P”) in and along the grain boundaries.

The microstructure of 5083 Al after consolidation and extrusion exhibited an average grain size of about 305 nm [70,71]. Inspection of the initial microstructure (Figures 10(a)) after annealing for 30 min at 673 K revealed the following observations:

- The grain size was estimated to be 440 ± 50 nm. Both SEM and TEM were used in measuring the grain size and the results were in good agreement. Careful TEM examination showed the presence of few grains of both nano size (<100 nm) and micron size.
- Nano-scale dispersion particles were mostly present in the interior of the grains and close to the grain boundaries (Figure 16). Some particles existed close to boundaries and few were found along boundaries. In general, the distribution of the particles was not uniform; the density of the particles could be fairly high in some local regions. Most of the particles were nearly spherical in shape. In their investigation on 5083 Al after milling, Tellkamp *et al.* [72] reported the presence of Mg_2_Si, Al_2_O_3_, and AlN.
- Dislocations were also found in few grains but the overall density was quite low.

**Figure 17:**
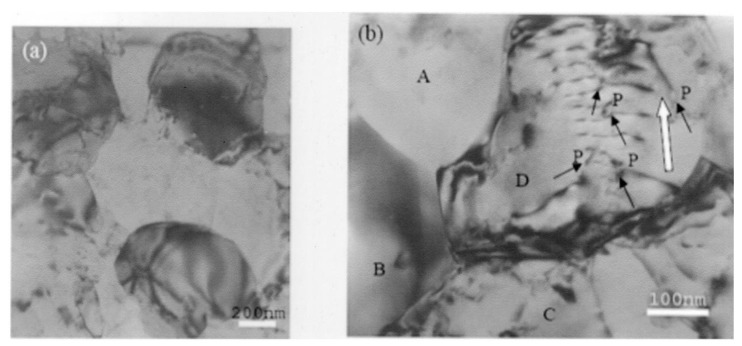
TEM micrograph of crept samples showing (**a**) dislocation free grains (T = 648 K and *τ* = 23 MPa); (**b**) the presence of dislocations in only few grains (T = 623 K and *t* = 19.2 MPa).

Following creep testing, a TEM examination of creep microstructure revealed the following observations:

- Dislocations are present in only few grains while most of the grains, especially the small ones, were found to have extremely low dislocations density (Figure 17(a)).
- Dislocations exhibited the following configurations: (i) most of them are parallel (Figure 17(b)); (ii) they tended to be long and curved; and (iii) arrays of dislocations or simple boundaries that sometimes extended from a boundary to another are noted in large grains (d > 500 nm).
- Many of the dislocations noted in the interiors of grains are attached to dispersion particles (Figure 17(b)). The attachment configurations were reported for other materials containing dispersion particles [73,74,75]. In particular, the configuration in Figure 8(b) shows a group of essentially parallel dislocations. While moving in the interior of the grain toward a boundary, these dislocations encountered a particle.

**Figure 18:**
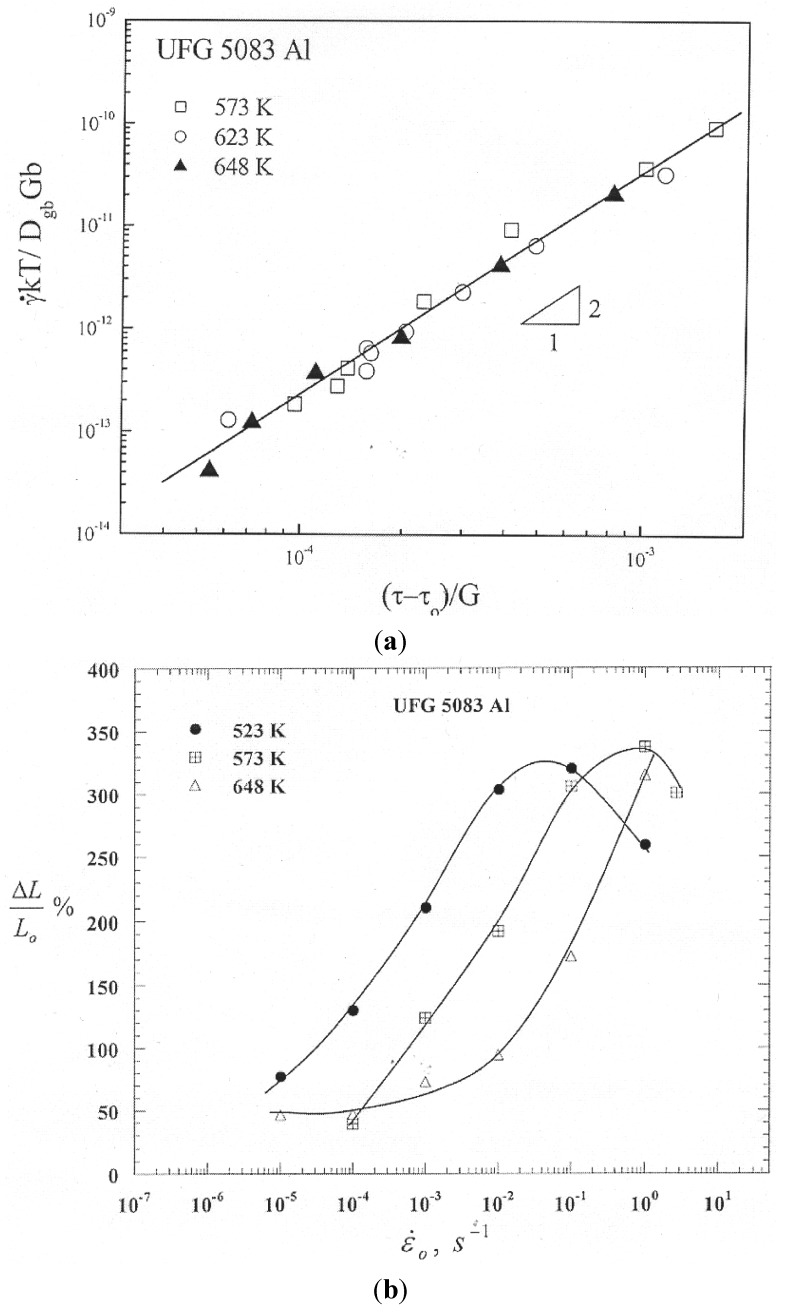
(**a**) Normalized strain rate *vs.* normalized effective stress; (**b**) Ductility in UFG 5083Al.

Consideration of the mechanical and substructural data on 5083 Al that was prepared by cryomilling followed by consolidation led to the following findings [71]:

- The creep characteristics of 5083 Al were consistent with those associated with HSR superplasticity. This consistency is demonstrated by the following: (i) a true stress exponent of about 2 (Figure 18(a)), (ii) optimum elongations of about 350 % at strain rates higher than 0.1 s^−1^ (Figure 18(b)), (iii) an equiaxed grain structure after extensive creep deformation, and (iv) evidence of the occurrence of boundary sliding and its features such as offsets, voids, striated bands, rotation of grains. The present study provides the first detailed evidence for HSR superplasticity in an Al alloy that is prepared by spraying followed by cryomilling and consolidation.
- The superplastic behavior of UFG 5083 Al, like that reported for micrograin superplastic alloys such as Zn-22% Al, exhibits a threshold stress. The presence of such threshold stress provides an explanation for: (i) the continuous increase in both the apparent stress exponent and the apparent activation energy with decreasing applied stress, and (ii) the loss in ductility at low strain rates.
- The process of gas atomization and cryomilling, unlike ECAP, does not require the addition of elements such as Sc to 5083 Al in order to suppress grain growth during superplastic deformation. Nano-scale particles are naturally introduced in 5083 Al during the process of gas atomization and cryomilling.
- Dispersion particles play the following roles: (i) providing thermal stability by suppressing grain growth, (ii) interacting with dislocations during their glide motion in the blocking grains or their motion in boundaries to produce sliding, (iii) serving as a possible source of the threshold stress, and (iv) acting as sites of cavitation and hence leading to premature facture.

### 2.7. Superplasticity in Nanocrystalline Materials

Nanocrystalline (nc) materials are characterized by grain sizes ≤ 200 nm. Because of this characteristic, grain boundaries, junction lines, and nodes have significant volume fractions—substructural features that can influence properties far more strongly than in conventional materials [76,77,78].

According to information on micrograin superplasticity, as the grain size decreases from micrometer to nanometer, it is expected that the superplastic region (Region II) would be transposed to high strain rates or observed at low temperatures such as room temperature, *i.e.*, high strain rate and/or low temperature superplasticity is possible. Despite this attractive possibility and its implications in terms of the potential for industrial adaptation of superplastic forming, attempts made so far to explore the occurrence of superplasticity have been unsuccessful.

One possible reason for the difficulty of observing superplastic flow in nc-materials is the occurrence of grain growth: it is not possible to obtain low-temperature superplasticity in pure metals in which grain sizes are less than 100 nm since the advantage of observing superplastic flow at low temperatures is neutralized by the onset of significant grain growth in this temperature range. 

**Figure 19:**
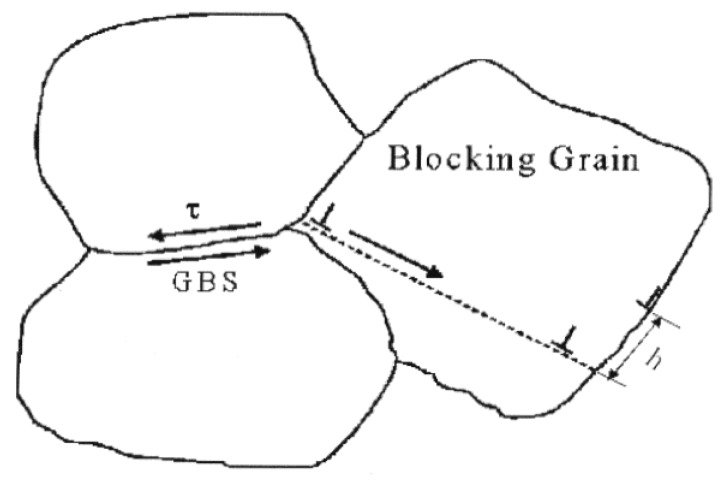
Schematic diagram for the model of dislocation-accommodated boundary sliding, showing that as a result of boundary sliding, dislocations are generated at a triple junction and then traverse the grain to the opposite grain boundary where they climb and are annihilated.

A second reason can be inferred from the rate controlling mechanism for deformation in nc-materials. Recently, a new model for deformation in nc-materials has been developed [79]. The development of the model was based on the concept that plasticity in nc-materials is the result of grain boundary sliding accommodated by the generation and motion of dislocations under local stresses, which are higher than applied stresses due to the development of stress concentrations. Specifically, it has been assumed that as a result of sliding of a group of grains, the shear stress becomes concentrated at any grain, triple point, or protrusion that obstructs motion of this group; that this high local stress can then generate dislocations in the blocking grain (or initiate voids); and that the generated dislocations move one by one to the opposite boundary where they climb to their annihilation sites (no dislocation-ups). For illustration, see Figure 19. By postulating that the creep rate, $\dot{\gamma }$ is governed by the time for the climb of a dislocation along the boundary until annihilation occurs, the following rate-controlling equation was derived [79]:

(7) $$\dot{\gamma }=9{\left(\frac{b}{d}\right)}^{3}\frac{D_{gbo}}{b^{2}}exp(\frac{-Q_{gb}}{RT})exp(\frac{\tau v}{kT})-1]$$

where *b* is the Burgers vector, *D_gbo_* is the frequency factor for grain boundary diffusion, and *Q_gb_* is the activation energy for grain boundary diffusion. The stress exponent for creep, *n*, as estimated from Equation (9), exhibits high and variable values; for the applicable range of stresses, *n* > 5. Accordingly, it is expected that ductility in nc-materials would be much lower than those characterizing micrograined superplastic alloys for which *n* = 2, since ductility depends on 1/*n* (*n* = 1/*m*) as shown by the following equation [80]:

(8) $$e_{f}\%=[exp(C/(n-1))]-1]\;x$$

where C = (*n* − (*1*/*n*)) *ln* (*400*/n); *n* = *1*/*m*. As shown in reference [6], combining Equation (9) and (10) leads to predicting that ductility is low, in the range 2–10%. From a microstructural point of view, the low ductility can be explained in terms of the details of the concept of dislocation-accommodated boundary sliding [79]. According to the concept, dislocations are generated under the local stresses and move in the blocking grains to boundaries where they are annihilated. This scheme results in the absence of dislocation accumulation and interactions, *i.e.*, nanograins are not able to sustain arrays of dislocations. This leads to loss of work hardening, which in turn results in low ductility. 

## 3. Conclusions

Over the past four decades, significant progress has been made in rationalizing micrograin superplasticity. Such progress is reflected in the following findings:

(1) For micrograined superplastic alloys (d < 10 µm), such as Zn-22 pct Al, that display superplastic behavior (tensile elongation > 500%) and whose creep behavior often exhibits a sigmoidal relationship between stress and strain rate, the low-stress region of such a relationship (Region I) as well as cavitation are absent when impurity level becomes less than 6ppm. By contrast, when impurities are present, Region I becomes well defined, a threshold stress for superplastic behavior exists, and cavities tend to align along the tensile axis (cavity stringers). These effects, and others, are most probably a consequence of impurity segregation at boundaries,

(2) The effect of impurities level on superplastic deformation and cavitation corresponds well with the effect of impurity level on the contribution of boundary sliding to total strain at low elongations (20–30%). This correspondence reflects an interaction between two roles played by boundaries during superplastic deformation. The first role is related to the ability of boundaries to contribute to deformation through the process of boundary sliding while the second role pertains to their ability to serve as favorable sites for the accumulation of impurities, *i.e*., boundary segregation. Under the present experimental conditions, boundary sliding is an important feature of the deformation process that controls steady-state superplastic flow. 

(3) The correlation between impurity level and the sliding contribution at low strain rates is most probably a reflection of the influence of impurities on accommodation processes for boundary sliding such as boundary migration and lattice dislocation motion

(4) By introducing nanometer-scale dispersion particles in Zn-22% Al via cryomilling, it was demonstrated that lattice dislocation activity occurs during the superplastic deformation. This finding when combined with earlier observations such as the insignificant contribution of diffusional creep to deformation in Region II and the absence of cavitation in high purity Zn-22% Al, signifies that lattice dislocation activity serves as an accommodation process for boundary sliding.

(5) Our understanding of micrograin superplasticity in model materials (1 μm < *d* < 10 μm) has led to a major advance: Achieving high strain rate superplasticity in ultrafine grained materials (200 μm < *d* < 1 mm) that is beneficial to producing Al alloys for commercial applications. Two severe plastic deformation techniques, ECAP and cryomilling, have been successful in producing ultrafine grained materials that exhibit superplastic behavior. 

(6) Attempts to observe superplasticity in nanocrystalline materials have so far been unsuccessful. Two factors may be responsible: (a) significant grain growth occurs during deformation; and (b) the proposed deformation mechanism controlling deformation in nanocrystalline materials predicts a stress exponent, n > 5; for micrograin superplasticity, n~2.
